# Role of the RNA-Binding Protein Nrd1 in Stress Granule Formation and Its Implication in the Stress Response in Fission Yeast

**DOI:** 10.1371/journal.pone.0029683

**Published:** 2012-01-19

**Authors:** Ryosuke Satoh, Akitomo Tanaka, Ayako Kita, Takahiro Morita, Yasuhiro Matsumura, Nanae Umeda, Makoto Takada, Sachiko Hayashi, Tokio Tani, Kaori Shinmyozu, Reiko Sugiura

**Affiliations:** 1 Laboratory of Molecular Pharmacogenomics, School of Pharmaceutical Sciences, Kinki University, Osaka, Japan; 2 Japan Society for the Promotion of Science, Tokyo, Japan; 3 Department of Biological Sciences, Graduate School of Science and Technology, Kumamoto University, Kumamoto, Japan; 4 Proteomics Support Unit, RIKEN Center for Developmental Biology, Hyogo, Japan; University of Cambridge, United Kingdom

## Abstract

We have previously identified the RNA recognition motif (RRM)-type RNA-binding protein Nrd1 as an important regulator of the posttranscriptional expression of myosin in fission yeast. Pmk1 MAPK-dependent phosphorylation negatively regulates the RNA-binding activity of Nrd1. Here, we report the role of Nrd1 in stress-induced RNA granules. Nrd1 can localize to poly(A)-binding protein (Pabp)-positive RNA granules in response to various stress stimuli, including heat shock, arsenite treatment, and oxidative stress. Interestingly, compared with the unphosphorylatable Nrd1, Nrd1^DD^ (phosphorylation-mimic version of Nrd1) translocates more quickly from the cytoplasm to the stress granules in response to various stimuli; this suggests that the phosphorylation of Nrd1 by MAPK enhances its localization to stress-induced cytoplasmic granules. Nrd1 binds to Cpc2 (fission yeast RACK) in a phosphorylation-dependent manner and deletion of Cpc2 affects the formation of Nrd1-positive granules upon arsenite treatment. Moreover, the depletion of Nrd1 leads to a delay in Pabp-positive RNA granule formation, and overexpression of Nrd1 results in an increased size and number of Pabp-positive granules. Interestingly, Nrd1 deletion induced resistance to sustained stresses and enhanced sensitivity to transient stresses. In conclusion, our results indicate that Nrd1 plays a role in stress-induced granule formation, which affects stress resistance in fission yeast.

## Introduction

Stress granules (SGs) are non-membranous cytoplasmic foci, composed of non-translating messenger ribonucleoproteins (mRNPs) that rapidly accumulate in cells exposed to a broad range of environmental stresses, including oxidative, genotoxic, hyperosmotic, or heat shock stresses [1], [2], [3]. Several components of SGs have been identified, including the related RNA-binding proteins TIA-1 and TIAR, poly(A)-binding protein (PABP), and translation factors such as eIF3, eIF4E, and eIF4G [4]. In mammalian cells, the key event leading to the formation of SGs is the stress-induced phosphorylation of the translation initiation factor eIF2α [5]. The assembly of SGs in response to the phosphorylation of eIF2α is dependent on TIA-1 and TIAR; thus, these proteins are key regulators of SG formation and assembly [5]. The structural domains of these proteins required for the assembly of SGs are the RNA recognition motifs (RRMs) at their N-termini and the prion-related domains at their C-termini. Identification of TIA-1 mRNA targets showed that this protein binds to a U-rich motif localizing preferentially to the 3′-untranslated regions of target genes [6].

Stress granules have been observed in yeast, such as fission yeast and budding yeast, protozoa and metazoa [1], [2], [3]. In budding yeast, the components and kinetics of SG assembly are extensively studied and although many components of SGs are highly conserved in this organism, stress-granule assembly and its composition can vary in a stress-specific manner in yeast [7]. Recently, some of the proteins that localize to SGs in fission yeast have been identified, including Vgl1, a multi-KH-type RNA-binding protein [8], and the role of PKA in the regulation of SGs has also been reported [9]. However, only a few players of the fission yeast SGs have been identified to date and the physiological significance of SGs in stress response has not been fully elucidated in this organism.

We previously identified Nrd1, an RRM-type RNA-binding protein, as a regulator of cytokinesis [10] by demonstrating that Nrd1 directly binds and stabilizes Cdc4 mRNA encoding a myosin light chain in fission yeast [10], in addition to its well-known role as a negative regulator of sexual differentiation [11], [12], [13]. We also demonstrated that the Pmk1 MAPK-dependent phosphorylation negatively regulates Nrd1 activity and cytokinesis through myosin mRNA stability [10]. Intriguingly, Nrd1 shares significant sequence similarity and a common preferred RNA-binding sequence (UCUU) with TIA-1 or TIAR [10]. This prompted us to investigate whether Nrd1, like TIA-1/TIAR, plays a role in SG assembly in response to adverse environmental stimuli. In this study, we showed that Nrd1 forms RNA granules in response to various stresses. Notably, Nrd1 localization to stress granules is modulated by phosphorylation and Cpc2, which is a RACK homologue in fission yeast. In addition, deletion of Nrd1 affects the sensitivity to these stresses in fission yeast. We propose that Nrd1 is a key component of SGs coordinating stress responses and SG formation.

## Materials and Methods

### Strains, Media, and Genetic and Molecular Biology Methods

*Schizosaccharomyces pombe* strains used in this study are listed in Table 1. The complete medium (yeast extract-peptone-dextrose [YPD]), (yeast extract with supplements [YES]) and the minimal medium (Edinburgh minimal medium [EMM]) have been described previously [14], [15]. Standard genetic and recombinant DNA methods [14] were used except where otherwise noted. PCR-based genomic epitope tagging was performed using standard methods [16]. In all cases, proteins were C-terminally tagged with GFP, YFP, or tdTomato and expressed from the respective endogenous loci.

**Table 1:** *Schizosaccharomyces pombe* strains used in this study.

Strain	Genotype	Reference
**1243**	*h^−^ ade6-M210 leu1-32 ura4-D18 eIF2α-S52A::ura4^+^*	Tvegård *et al.*, 2007 [32]
**AN102**	*h^−^ leu-32 ura4D-18 cpc2::KanMX6 cpc2-GFP::leu1^+^ pyp2-13myc::ura4^+^*	Núñez *et al.*, 2009 [27]
**HM123**	*h^−^ leu1-32*	Our stock
**HT201**	*h^90^ ade6-M210 leu1-32 ura4-D18 cpc2::ura4^+^*	Jeong *et al.*, 2004 [13]
**KAY296**	*h^−^ ade6-M216 leu1-32 ura4-D18 hri1::ura4^+^ hri1::leu1^+^ gcn2::ura4^+^*	Udagawa *et al.*, 2008 [33]
**KP456**	*h^−^ leu1-32 ura4-D18*	Our stock
**KP616**	*h^−^ leu1-32 ura4-D18 nrd1::ura4^+^*	Our stock
**KP928**	*h^+^ his2 leu1-32 ura4-D18*	Our stock
**SP755**	*h^−^ leu1-32 ura4-D18 cpc2::ura4^+^*	This study
**SP1074**	*h^−^ leu1-32 cpc2-GFP::KanMX6*	This study
**SP1263**	*h^−^ leu1-32 nmt1-pabp-YFP-FLAG-6His::leu1^+^*	This study
**SP1265**	*h^−^ leu1-32 ura4-D18 nrd1::ura4^+^ nmt1-pabp-YFP-FLAG-6His::leu1^+^*	This study
**SP1341**	*h^−^ leu1-32 ura4-D18 cpc2::KanMX6 cpc2-GFP::leu1^+^*	This study
**SP1558**	*h^−^ leu1-32 nrd1-GFP::KanMX6*	This study
**SP1576**	*h^−^ nrd1-tdTomato::HphMX6 pabp-GFP::KanMX6*	This study
**SP1651**	*h^−^ ade6-M210 leu1-32 ura4-D18 eIF2α-S52A::ura4^+^ nrd1-GFP-KanMX6*	This study
**SP1652**	*h^−^ ade6-216 leu1-32 ura4-D18 hri1::ura4^+^ hri2::leu1^+^ gcn2::ura4^+^ nrd1-GFP-KanMX6*	This study
**SP1660**	*h^−^ leu1-32 ura4-D18 pmk1::ura4^+^ nrd1-GFP::KanMX6*	This study
**YO15**	*h^90^ ade6-M210 leu1-32 ura4-D18 msa2:3HA-KanMX6 cpc2-GFP::KanMX6*	Jeong *et al.*, 2004[13]

### Protein Expression, Site-Directed Mutagenesis, and Phosphorylation Assays

For protein expression in yeast, the thiamine-repressible *nmt1* promoter was used [17]. Expression was repressed by the addition of 4.0 µg/ml thiamine to EMM and was induced by washing and incubating the cells in EMM lacking thiamine. The GST-, the YFP-, the mCherry-, or the GFP-fused gene was subcloned into the pREP1, or pREP2 vectors.

### Growth Conditions and Stress Treatment

Unless otherwise stated, cells were cultivated at 27°C in EMM, or YES rich medium [14]. Prior to stress treatment, the cells were grown to mid-log phase (OD_660 nm_ = 0.5). Heat shock was imposed by transferring the culture tubes to a water bath at 42°C for the indicated time. To the culture medium, 200 mM sodium arsenite (Wako) stock solution, 3.0% H_2_O_2_ (Nakarai Tesque) stock solution, 1.0 M CdCl_2_ (Nakarai Tesque) stock solution, or 4.0 M KCl (Nakarai Tesque) stock solution was added at the indicated concentrations. After each stress treatment, the culture medium was chilled in ice water for 5 minutes. The cells were harvested by brief centrifugation at 4°C.

### Fluorescence *In Situ* Hybridization

The cells were grown to the mid-log phase in synthetic minimal medium at 26°C and exposed to heat shock at 42°C for 20 min or treated with 2.0 mM sodium arsenite for 40 min. Fluorescence *in situ* hybridization was then performed according to a previously published procedure with slight modifications [18]. Hybridization with a biotin-labeled oligo dT probe (50-mer) was performed at 37°C. After hybridization, the cells were washed and treated with fluorescein isothiocyanate (FITC)-conjugated avidin for 30 min at room temperature [19]. Photos of hybridized cells were acquired using Keyence BIOREVO BZ-9000.

### Microscopy and Miscellaneous Methods

Light microscopy methods, such as differential interference contrast (DIC) and fluorescence microscopy, were performed as described [20]. Cell extract preparation and immunoblot analysis were performed as previously described [21].

### Image Quantification

The quantification of stress granule foci was done for 3 individual datasets which summed up to 150 counted cells.

## Results

### Nrd1 Localizes to Stress-Induced Granules

**Figure 1:**
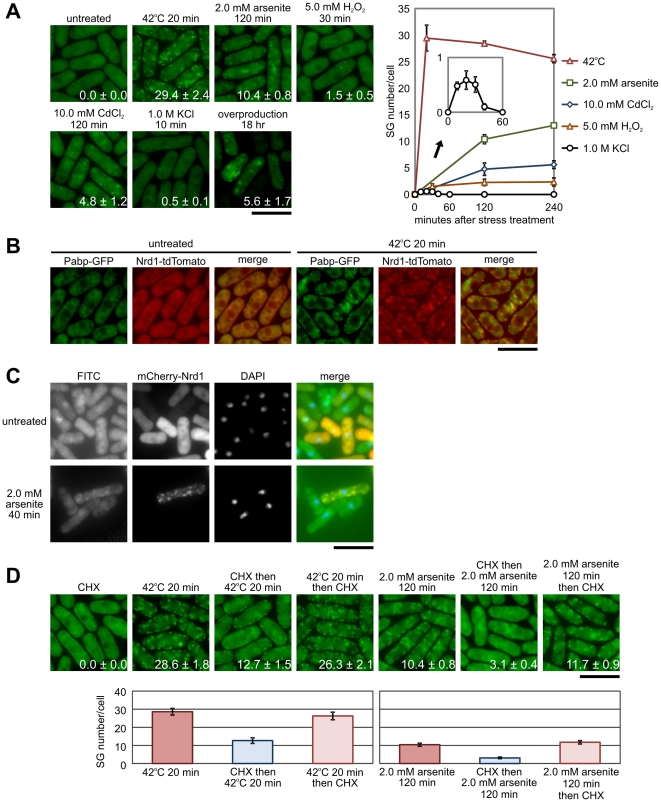
Nrd1 localizes to stress granules under various environmental stresses. (A) Analysis of Nrd1-GFP localization under stress. Localization of Nrd1-GFP in living cells grown at 27°C (untreated) after a shift to 42°C for 20 min (42°C 20 min) and after exposure to 2.0 mM arsenite (2.0 mM arsenite 120 min), 5.0 mM H_2_O_2_ (5.0 mM H_2_O_2_ 30 min), 10.0 mM CdCl_2_ (10.0 mM CdCl_2_ 120 min), or 1.0 M KCl (1.0 M KCl 10 min) for the times indicated. Wild-type (wt) cells transformed with pREP1-GFP-Nrd1 were grown in EMM (thiamine-free medium) for 18 h to induce overproduction of GFP-Nrd1 (overproduction 18 hr). Bar, 10 µm. The number in the picture indicates the SG number/cell in each experiment. Right panel: Quantitative analysis of the number of SGs/cell on each stress. Graph depicting the number of stress granules per cell formed before (untreated) and after each condition as indicated in Figure 1(A) plotted against time after exposure to each stress and the *inset* is a magnification of the results obtained on KCl treatment. (B) Co-localization of Nrd1 with poly(A)-binding protein (Pabp). Merged image of fluorescence micrographs showing Pabp-GFP (green) and Nrd1-tdTomato (red) in untreated cells and after a 20-min incubation at 42°C. Bar, 10 µm. (C) Fluorescence micrographs of the wild-type cells expressing mCherry-tagged Nrd1 grown at 26°C (untreated); and these were subjected to *in situ* hybridization with a digoxigenin-labeled oligo (dT)_50_ probe after a 40-min exposure to 2.0 mM arsenite (2.0 mM arsenite 40 min). The hybridized probe was detected by treatment with mouse anti-digoxin antibody, followed by a fluorescein-conjugated goat anti-mouse IgG antibody (FITC). Nrd1 was detected using mCherry fluorescence (mCherry-Nrd1). Nuclei are counterstained using DAPI dye (DAPI). Bar, 10 µm. (D) Cycloheximide (CHX) prevents the formation of heat-shock- and arsenite-induced Nrd1 granules. Fluorescent images of cells expressing Nrd1-GFP incubated (from left to right) at 27°C with 100 µg/ml CHX for 30 min (CHX); at 42°C for 20 min; pre-incubated with CHX for 30 min at 27°C followed by 20-min incubation at 42°C (CHX then 42°C 20 min); 20-min incubation at 42°C followed by CHX incubation (42°C 20 min then CHX); with 2.0 mM arsenite for 120 min at 27°C; pre-incubated with CHX for 30 min followed by 120-min incubation with 2.0 mM arsenite; and 120-min pre-incubation with arsenite followed by 30-min incubation at with CHX. Bar, 10 µm. Lower panel: Graph depicting the number of stress granules per cell in each condition plotted against time after exposure to each stress.

To examine the possible functions of Nrd1 in SG formation, we determined the subcellular localization of Nrd1 in response to environmental stimuli. In unstressed cells, chromosomally integrated GFP-tagged Nrd1 was localized diffusely in the cytosol (Figure 1A; untreated). Upon exposure to thermal stress for 20 min, small patches of granule-like structures were clearly observed throughout the cytoplasm (Figure 1A; 42°C; 20 min). Intriguingly, exposure of cells to arsenite, a chemical well-known to induce SGs in mammalian cells also induced the Nrd1-positive granules (Figure 1A; 2.0 mM arsenite; 120 min). In addition, as shown in Figure 1A, Nrd1-positive granules are also formed in cells treated with H_2_O_2_ (5.0 mM H_2_O_2_; 30 min), CdCl_2_ (10.0 mM CdCl_2_; 120 min), and hyperosmotic stress (1.0 M KCl; 10 min).

To precisely define the conditions of Nrd1 granules formation at each condition, we have performed a quantitative analysis of Nrd1-positive granule number/cell for each stress. The results showed that heat shock induced the strongest stimulation of Nrd1-positive granules (Figure 1A; right panel). Arsenite also induced the second strongest effect on Nrd1 -granule formation. In contrast, H_2_O_2_ and CdCl_2_ exerted a relatively weak effect on granule formation as compared with heat and arsenite stress (Figure 1A; right panel). Regarding the kinetics of Nrd1 granule formation, heat shock caused a rapid emergence of granules, whereas oxidative stress induces gradual appearance of granules (Figure 1A; right panel). Notably, regarding the intensity of granule formation, KCl treatment exerted a very weak effect (0.5±0.1% at 10 min) as compared with the other stresses. In addition, the kinetics of Nrd1 granule formation in response to KCl treatment was different in comparison to other stimuli in that it induced granule formation very quickly, with its peak at 20 min (Figure 1A; right panel). The number of Nrd1 granules per cell decreased and reached zero after a 60-min exposure of the cells to KCl, whereas the cells still continued to induce Nrd1 granule formation at this time point when exposed to other stimuli (Figure 1A). We also examined the effect of Nrd1 overproduction on granule formation. For this, we expressed GFP-Nrd1 under the control of the *nmt1* promoter, which is repressed in the presence of thiamine. Overproduction of Nrd1 stimulated Nrd1-positive granule formation in the absence of stress (Figure 1A; overexpressed for 18 h).

In mammalian cells and in both budding and fission yeasts, poly(A)-binding protein (Pabp) is an SG marker [5], [9], [22]. Therefore, we used GFP-tagged Pabp, the major poly(A)-binding protein of *S. pombe*, to examine its co-localization with Nrd1. For this experiment, we constructed a strain expressing Nrd1 protein tagged with tdTomato and Pabp protein tagged with GFP expressed from their respective endogenous loci. In unstressed cells, both Nrd1 and Pabp were localized diffusely in the cytosol (Figure 1B; untreated). The fluorescence of Nrd1 and Pabp largely co-localizes in cytoplasmic foci on heat stress (Figure 1B, 42°C 20 min).

To determine whether the Nrd1-positive granules actually contain mRNA, we visualized cellular poly(A)^+^ RNA by *in situ* hybridization *in vivo* in cells expressing mCherry-tagged Nrd1 and compared the subcellular localization of poly(A)^+^ RNA and Nrd1. Notably, arsenite treatment induced near-complete co-localization of poly(A)^+^ RNA and Nrd1 in the cytoplasm (Figure 1C). Thus, Nrd1-positive granules correspond to SGs comprising of ribonucleoprotein complexes in *S. pombe*.

### Effects of Cycloheximide on Nrd1-positive RNA Granules

We investigated whether cycloheximide (CHX) could prevent Nrd1-positive RNA granule formation in *S. pombe*, since RNA granule formation by external stress can be blocked through inhibition of protein synthesis by CHX in mammalian cells and in fission yeast [9], [23]. The localization of Nrd1 was not affected upon CHX treatment (Figure 1D; CHX). Pre-treatment with CHX largely prevented the formation of Nrd1-positive granules induced by heat shock or arsenite treatment (Figure 1D; CHX then 42°C for 20 min, CHX then arsenite for 120 min). It should be noted that the addition of CHX after 20-min exposure to heat shock or 120-min exposure to arsenite failed to block granule formation (42°C for 20 min then CHX, arsenite for 120 min then CHX). We also counted the number of SG per cell to quantitatively assess the effect of CHX on Nrd1 granule assembly, and the results confirmed the above findings (Figure 1D; lower panel). The fact that the known inhibitor of SG formation was effective in preventing Nrd1 granule formation suggests that the Nrd1 granules correspond to SGs in mammals and that this is a biologically regulated process.

### Nrd1 Localization to Stress Granules is Modulated by Phosphorylation Under Certain Stresses

**Figure 2:**
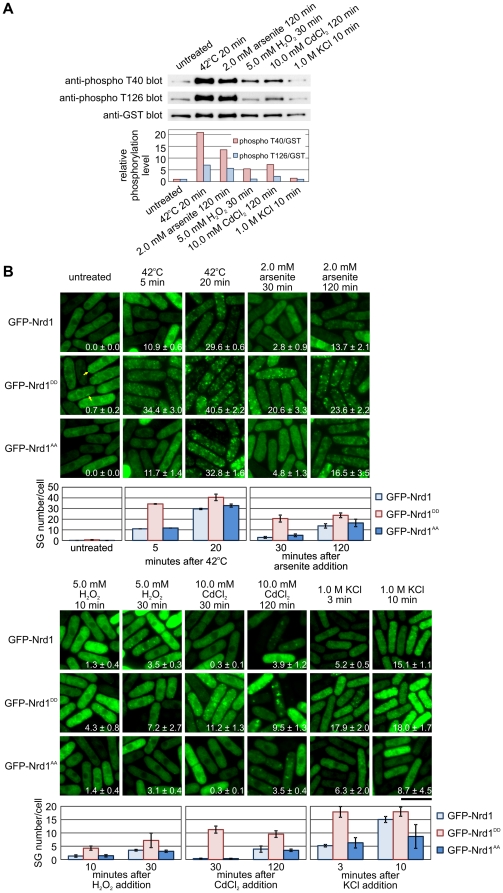
Nrd1 localization to stress granules is modulated by phosphorylation under certain stresses. (A) Nrd1 is phosphorylated in response to various environmental stresses. Wild-type cells expressing GST-tagged Nrd1 were grown in EMM at 27°C (untreated). Cells were treated with heat shock (42°C 20 min), 2.0 mM arsenite for 120 min, 5.0 mM H_2_O_2_ for 30 min, 10.0 mM CdCl_2_ for 120 min, or 1.0 M KCl for 10 min. Proteins bound to glutathione sepharose were analyzed by SDS-PAGE and immunoblotting using anti-phospho Nrd1 T40 (anti-phospho T40 blot), anti-phospho Nrd1 T126 (anti-phospho T126 blot), and anti-GST (anti-GST blot) antibodies. Lower panel: Quantification of phosphorylation levels of Nrd1 (T40) and (T120) against GST-Nrd1 protein levels under various stresses as shown in Figure 2(A). (B) Phosphorylation-dependent localization of Nrd1. Fluorescent images of the wild-type cells expressing GFP-tagged Nrd1, Nrd1^DD^, or Nrd1^AA^ grown in normal medium (EMM+thiamine) at 27°C (untreated), or exposed to various stresses under the conditions indicated. The number in the picture indicates SG number/cell in each condition. Bar, 10 µm. Lower panel: Graphs show the number of stress granules per cell from each strain.

Our previous results showed that heat-shock induced Pmk1 MAPK-dependent phosphorylation of Nrd1 [10]. This prompted us to investigate whether various stimuli that induced Nrd1-positive SG formation also affect the phosphorylation levels of Nrd1. We utilized anti-phospho Nrd1 T40 antibodies and anti-phospho Nrd1 T126 antibodies that recognize the phosphorylated Thr40 or Thr126, respectively [10]. As shown in Figure 2A, the levels of Nrd1 phosphorylation were markedly increased in response to heat shock (42°C for 20 min) and arsenite treatment (2.0 mM arsenite for 120 min). In addition, H_2_O_2_, and CdCl_2_ treatment moderately induced the phosphorylation of Nrd1 at T40, whereas KCl treatment, did not significantly affect the phosphorylation levels of Nrd1. It should be noted that the strength of Nrd1 phosphorylation by each stress roughly parallels that of SG formation as judged by the number of SG/cell treated by each stress as shown in Fig. 1A. Thus, the distinct kinetics of SG assembly on exposure to KCl and the lack of Pmk1 action on the Nrd1 substrate on KCl may be because of negative feedback regulation, such as induction of phosphatases that dephosphorylate Pmk1 MAPK. Our previous reports showed that KCl treatment activates a broad range of signaling pathways including the p38/Spc1 MAPK pathway, which induces Ptc1/Ptc3 phosphatase to inactivate both Spc1 and Pmk1 MAPK [24]. In addition, it should be noted that H_2_O_2_ and CdCl_2_ treatment promote Nrd1 phosphorylation predominantly at T40 (Figure 2A). It can be interpreted that the selectivity of MAPK and/or phosphatases that inactivate Nrd1 may be different in these two threonine residues.

We then investigated whether phosphorylation enhanced Nrd1 translocation to SGs in response to stimuli. For this, we expressed the GFP-fused phosphorylation-mimic Nrd1^DD^, wherein T40 and T126 had been replaced with aspartic acid, and unphosphorylatable Nrd1^AA^, wherein T40 and T126 had been replaced with alanine [10], and compared their localization with that of the wild-type GFP-Nrd1. In untreated cells (27°C), wild-type Nrd1 and Nrd1^AA^ were localized diffusely in the cytosol, whereas a small portion of Nrd1^DD^ accumulated as patch-like structures (Figure 2B; arrows). Upon heat shock (42°C), Nrd1^DD^ markedly translocated to the stress granules after 5 min (34.4±3.0%), whereas approximately 10% of the wild-type Nrd1 and Nrd1^AA^ translocated to the granules (Figure 2B; 42°C for 5 min). Nrd1^AA^ granules and the wild-type Nrd1 granules displayed almost similar kinetics in response to heat stress (Figure 2B: Nrd1^AA^). It should be noted that the Nrd1^DD^-positive granules were more distinct than the granules associated with the wild-type Nrd1 in the same timeframe after heat shock (Figure 2B; 42°C for 20 min). In addition, Nrd1^DD^ translocated to the granules 30 min after arsenite treatment, 10 min after H_2_O_2_ stimulation, 30 min after CdCl_2_ treatment and 3 min after KCl treatment, whereas most of the wild-type Nrd1 and Nrd1^AA^ remained in the cytoplasm at these time points (Figure 2B). Similar to heat shock, wild-type Nrd1 and Nrd1^AA^ translocated to the granule structures after longer exposure to these stimuli (Figure 2B; arsenite 120 min). We also scored the SG number/cell in cells expressing different Nrd1 mutants, and the quantitative data showed that phosphorylation enhanced the rate of translocation of Nrd1 to SGs (Figure 2B). It should be mentioned that the effect of KCl on SG assembly was greater as compared with the data shown in Figure 1A. The difference may be because of the strains used in each experiment in which the endogenous Nrd1 protein was visualized in Figure 1A, whereas the plasmid versions of Nrd1 were used in Figure 2B. Alternatively, the difference may be because of the media used in each experiment in which the endogenous protein expression was performed using rich YES media, whereas the plasmid expression was performed using EMM.

**Figure 3:**
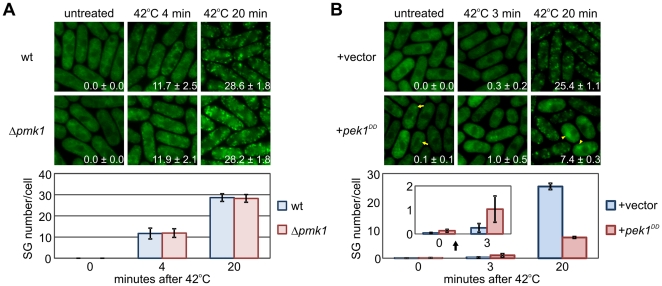
Effects of Pmk1 signaling on stress granule assembly. (A) Effects of Pmk1 deletion on SG assembly. Wild-type cells and Pmk1-deleted cells expressing Nrd1-GFP were grown in YES at 27°C (untreated). Cells were treated with heat shock (42°C for 4 min, or 20 min). Lower panel: Graphs showing the number of stress granules per cell from each strain. (B) Effects of Pmk1 hyperactivation on SG assembly. Wild-type cells expressing Nrd1-GFP were transformed with the constitutively active Pek1^DD^ or the control vector, and grown in EMM at 27°C (untreated). Cells underwent heat shock at 42°C for 3, or 20 min. Graphs showing the number of stress granules per cell from each strain. The inset is a magnification of the results obtained in each strain at 0 min and 3 min.

We next investigated the effects of Pmk1 absence or hyperactivation on SG formation under stress. The results revealed that in Pmk1-deleted cells, the kinetics and SG number/cell were almost the same as in the wild-type cells (Figure 3A). These data are consistent with the findings that non-phosphorylatable Nrd1^AA^ and the wild-type Nrd1 displayed similar kinetics of SG formation under stress (Figure 2B).

We also investigated effects of Pmk1 hyperactivation on SG formation by utilizing Pek1^DD^, the constitutively active MAPKK for Pmk1 MAPK. When Pek1^DD^ was overexpressed in cells expressing GFP-Nrd1, a subtle but clear population of the cells displayed SGs even in unstressed cells (Figure 3B, 0.1±0.1%, arrows). These data are consistent with the findings that the phosphorylation mimic Nrd1^DD^ also induced SG formation in unstressed cells (Figure 2B, GFP-Nrd1^DD^, 0.7±0.2%, arrows), although the effect is a little more prominent in Nrd1^DD^ overproduction. This difference can be explained by the recent findings that Nrd1 is phosphorylated not only by Pmk1 but also by Spk1 MAPK under nitrogen starved conditions [25]. In addition, when cells were exposed to a very short period of heat shock (3 min), Pek1^DD^-expressing cells displayed a greater number of SG appearance (1.0±0.5%) than that of the cells harboring the control vector alone (0.3±0.2%), suggesting that Pek1^DD^ expression stimulated the translocation of Nrd1 to SGs under heat shock (Figure 3B).

**Figure 4:**
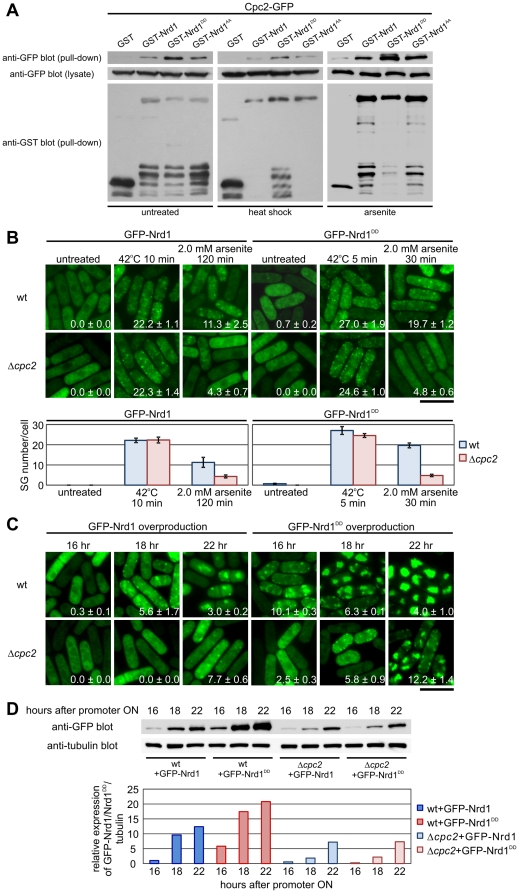
Cpc2 regulates arsenite-induced, but not heat shock-induced formation of Nrd1 granules. (A) Nrd1 binds to Cpc2 in a phosphorylation-dependent manner. Cells expressing endogenous Cpc2-GFP were transformed with plasmids harboring GST alone, GST-Nrd1, GST-Nrd1^DD^, or GST-Nrd1^AA^ and were grown to mid-log-phase in normal medium (EMM) at 27°C (untreated) and were exposed to a 10-min incubation at 42°C (heat shock), or 120-min incubation to 2.0 mM arsenite at 27°C (arsenite). Cell lysates (lysate) and proteins bound to glutathione sepharose (pull-down) were analyzed by immunoblotting using anti-GFP antibodies (lysates, and pull-down), and anti-GST antibodies (pull-down). (B) Effects of Cpc2 deletion on granule formation of Nrd1 and Nrd1^DD^ after heat shock or arsenite stress. GFP-Nrd1 or GFP-Nrd1^DD^ localization in the wild-type cells (wt) or Δ*cpc2* cells under the conditions indicated. Bar, 10 µm. Lower panel: Graphs showing the number of stress granules per cell from each strain. (C) Overproduction of Nrd1 induced dot-like structures in the absence of stress in a phosphorylation- and Cpc2-dependent manner. Wild-type (wt) or Δ*cpc2* cells transformed with pREP1-GFP-Nrd1 or pREP1-GFP-Nrd1^DD^ were grown in EMM (thiamine-free medium) for 16 h, 18 h, or 22 h to induce overproduction of GFP-Nrd1 or GFP-Nrd1^DD^. Bar, 10 µm. (D) Immunoblot of GFP-Nrd1 from each strain after each condition as indicated in Figure 4(C). Lower panel: Quantification of Nrd1 protein levels against tubulin at each condition as shown in Figure 4(D).

However, when Pek1^DD^-expressing cells were exposed to heat shock for 20 min, approximately half of the cells with a round morphology, because of Pek1^DD^ overproduction displayed the aggregation-like fluorescence of Nrd1 (Figure 3B, arrowheads). These structures may represent a consequence of the fusion of Nrd1 and/or SGs. Therefore, in this situation (heat shock for 20 min), the SG number/cell in Pek1^DD^-expressing cells was significantly lower (7.4±0.3%) than that of the cells harboring the control vector alone (25.4±1.1%). This unexpected result may reflect the above observation that the aggregation of Nrd1 observed in the Pek1^DD^-expressing cells apparently lowered the SG number/cell. Thus, Pek1^DD^ overproduction may lead to a similar situation wherein Nrd1 overproduction induced fusion of SGs and leads to an apparent decrease in the number of SG/cell (Figure 4C, 22 hr). Thus, the effects of Pek1^DD^ overproduction may be appropriate to assess by monitoring the size, but not SG number/cell.

### Phosphorylation-Dependent Binding of Nrd1 and Cpc2, a RACK Homologue in Fission Yeast

The above findings that Nrd1^DD^ translocated to the granule structures faster than the unphosphorylatable form of Nrd1 suggested the possibility that compared with the Nrd1^AA^, Nrd1^DD^ might have higher affinity to the component(s) of cytoplasmic granule structures. We then examined whether the phosphorylation of Nrd1 affects its ability to bind to Cpc2, the RACK1 orthologue in fission yeast, since Cpc2 was identified as a binding partner for Nrd1 [13] and Cpc2 has roles in translation, G2/M transition, and stress responses in fission yeast [26], [27]. We co-expressed various versions of GST-fused Nrd1 proteins in cells expressing the chromosomally tagged Cpc2-GFP from its endogenous promoter. As shown in Figure 4A, Cpc2-GFP bound more strongly to GST-Nrd1^DD^ as compared with the wild-type Nrd1 and Nrd1^AA^, both before (untreated) and after heat shock or arsenite treatment.

We next examined the functional relationship between Cpc2 and Nrd1 by assessing the effect of Cpc2 deletion on the localization of Nrd1 in SGs. In unstressed conditions, the localization of GFP-fused Nrd1 and Nrd1^DD^ proteins in wild-type cells and *cpc2* deletion cells (Δ*cpc2*) was indistinguishable (Figure 4B; untreated). Notably, in *cpc2* deletion cells, the assembly of Nrd1 and Nrd1^DD^ granules was markedly inhibited upon arsenite stress (Figure 4B; 2.0 mM arsenite). In contrast, Cpc2 did not affect Nrd1 and Nrd1^DD^ granule formation after heat shock, as *cpc2* deletion cells formed granules with normal kinetics and intensity under these circumstances (Figure 4B; 42°C). Thus, arsenite-induced Nrd1 localization to SGs is Cpc2-dependent. Quantitative analysis obtained after scoring the SG number/cell indicated that Cpc2 deletion did not significantly affect the localization of wild-type Nrd1 to SGs on heat shock. However, it affected the localization of Nrd1 and Nrd1^DD^ to SGs under arsenite treatment, although the effect is more prominent with Nrd1^DD^ (Figure 4B). It should be noted that the effect of Cpc2 deletion was greater in Nrd1^DD^ as compared with Nrd1, presumably because of the higher affinity between Cpc2 and Nrd1^DD^ as compared with Nrd1.

### Nrd1 Plays a Role in SG Formation

As described in Figure 1A, overproduction of Nrd1 stimulated SG formation in the absence of stress. Notably, granule formation was faster in the phosphorylated mutant version of Nrd1^DD^ (Figure 4C; GFP-Nrd1^DD^, overexpressed for 16 h) than in the wild-type Nrd1 (Figure 4C; GFP-Nrd1, overexpressed for 18 h). This finding also demonstrated that Nrd1-positive granule formation induced by its overproduction was modulated by phosphorylation.

It should be mentioned that significantly larger granules were induced by Nrd1^DD^ overproduction for more than 18 h as compared with those induced by various stimuli (Figure 4C; GFP-Nrd1, 22 h, GFP-Nrd1^DD^, 18 hr and 22 hr). These structures may represent a consequence of the fusion of Nrd1 and/or SGs. Therefore, in this situation, the SG number/cell in Nrd1^DD^-overexpressing cells for 18 hr and 22 hr was lower than that for 16 hr. In addition, the number of SG/cell in Cpc2-deletion cells was higher than that in the wild-type cells when Nrd1 or Nrd1^DD^ was overproduced for 22 hr. This unexpected result may reflect the aggregation of Nrd1 induced by its overproduction, which apparently lowered the SG number/cell. Thus, the effect of Nrd1^DD^ overproduction, similar to that of Pek1^DD^ may be appropriate to assess by monitoring the SG size, but not SG number/cell.

Interestingly, Cpc2 deletion also affected the formation of overproduction-induced Nrd1 granules, because in Δ*cpc2* cells, the formation of large granules of both wild-type GFP-Nrd1 and GFP-Nrd1^DD^ was clearly inhibited (Figure 4C; Δ*cpc2*). We then measured Nrd1 protein levels in both wild-type and Cpc2 deletion cells at the same time points shown in the above experiments. The results showed that protein levels of Nrd1 and Nrd1^DD^ were markedly lower in Cpc2 deletion cells (Figure 4D). The effect of Cpc2 deletion was more prominent in Nrd1^DD^, presumably because of its stronger physical interaction as shown in Figure 4A.

**Figure 5:**
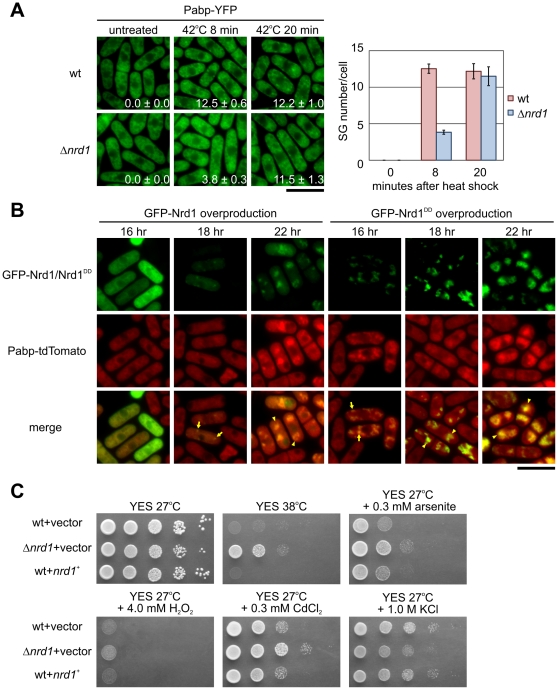
Nrd1 regulates stress granule assembly. (A) Effects of Nrd1 deletion on Pabp-positive granule formation after heat shock. Wild-type or the Δ*nrd1* cells expressing YFP-tagged Pabp were grown in normal medium or under the conditions indicated. Bar, 10 µm. Graphs showing the number of Pabp-positive granules per cell from each strain as a function of time. (B) Overproduction of Nrd1 induced Pabp-positive granules in the absence of stress. Cells expressing tdTomato-tagged Pabp transformed with pREP1-GFP-Nrd1 or pREP1-GFP-Nrd1^DD^ were grown in EMM (thiamine-free medium) for 16 h, 18 h, or 22 h to induce overproduction of GFP-Nrd1 or GFP-Nrd1^DD^. Bar, 10 µm. (C) Wild-type cells transformed with control vector or containing the *nrd1^+^* gene, or the Δ*nrd1* cells transformed with control vector, were grown in EMM+thiamine at 27°C and were then spotted onto YES plates, or spotted onto YES plates with 0.3 mM arsenite, 4.0 mM H_2_O_2_, 0.3 mM CdCl_2_, or 1.0 M KCl, and then incubated at temperatures indicated.

To determine the role of Nrd1 in SGs assembly in more detail, we examined the effect of Nrd1 absence on the assembly of SGs. For this, we compared the accumulation of the Pabp-positive granules after heat stress in wild-type cells and Nrd1 deletion cells. We found that the accumulation of the Pabp-positive granules (Pabp-YFP) in response to thermal stress was markedly inhibited in Nrd1 deletion cells than in the wild-type cells (Figure 5A). We performed a quantitative analysis of Pabp-YFP-positive granule numbers/cell in the wild-type and Nrd1 deletion cells under heat shock. The results showed that in Nrd1 deletion cells, the formation of the Pabp-positive granules was significantly inhibited at an 8 min exposure to high temperature, as the number of granules in Nrd1 deletion cells was about 30% of that in the wild-type cells (Figure 5A). However, after a heat shock treatment of 20 min, the formation of Pabp granules was not significantly affected in *nrd1*-deleted cells (Figure 5A).

Notably, we examined the effects of Nrd1 overproduction on Pabp -granules and the results showed that overproduction of GFP-Nrd1 and GFP-Nrd1^DD^ induced the Pabp-tdTomato-positive granule assembly (Figure 5B; arrows) and the aggregation/fusion of Pabp-granules, which co-localize with Nrd1 granules (Figure 5B; arrowheads). The aggregation of Pabp-granules was not observed in the cells overexpressing GFP vector alone (data not shown). These results demonstrated the important role of Nrd1 in SG formation.

SG assembly usually requires the stress-induced phosphorylation of the translation initiation factor eIF2α [28]. An allele expressing a phosphomimetic version of eIF2α is sufficient to induce SGs, whereas the expression of a mutant, unphosphorylatable version of eIF2α blocks SG formation upon stress [5]. In fission yeast, the kinetics of the appearance of RNA granules after hyperosmotic stress was slower in *eIF2α-S52A* cells than in wild-type cells [9]. To characterize the role of Nrd1 in SG formation and its relationship with eIF2α, we examined the effect of the *eIF2α-S52A* mutation on Nrd1 localization after stress. We observed dot-like structures of the endogenous Nrd1-GFP protein in *eIF2α-S52A* cells (*eIF2α-S52A*) after heat shock and arsenite treatment with kinetics similar to that observed in the wild-type cells (Figure S1). In addition, we examined the localization of Nrd1-GFP in triple mutants of eIF2α kinases (Δ*gcn2*Δ*hri1*Δ*hri2*), and found that the Nrd1 localization in the triple eIF2α kinase mutants was not distinguishable from that of the wild-type cells (Figure S1). Taken together, these results suggest that Nrd1 is involved in stress-induced granule assembly independent of the phosphorylation of eIF2α.

### Nrd1 Deletion Confers Resistance to Various Stresses

To test whether the disassembly of SGs (as shown above) caused by Nrd1 deletion would alter stress sensitivity, we exposed the Nrd1 deletion cells to heat, arsenite, CdCl_2_, H_2_O_2_, and KCl. Surprisingly, Nrd1 deletion cells were highly resistant to the high temperatures of 38°C, a temperature at which the wild-type cells barely grew (Figure 5C). While it is clear that Nrd1 deletion enhances cell tolerance against thermal stress, cell viability under arsenite or CdCl_2_ is only slightly enhanced, by less than an order of magnitude. No difference in growth can be observed in the presence of H_2_O_2_ (Figure 5C). Nrd1 deletion cells did not show resistance to KCl, and instead, the growth of Δ*nrd1* cells was slightly slower than that of the wild-type cells (Figure 5C; 1.0 M KCl). These results may be related to the stress-dependent nature of Nrd1 phosphorylation as shown in Figure 2A, wherein heat shock induced the strongest phosphorylation, followed by arsenite; CdCl_2_ and H_2_O_2_ give only a modest induction of Nrd1 phosphorylation, and KCl barely induced phosphorylation of Nrd1. The strength of Nrd1 phosphorylation by each stress roughly parallels that of SG formation as judged by number of SG/cell treated by each stress (Figure 2B). Therefore, Nrd1 deletion cells were more tolerant of thermal stress by delaying SG formation. The degree of tolerance to each stress associated with Nrd1 deletion may be the reflection of the avoidance of SG formation by Nrd1 deletion. Alternatively, Nrd1 may possess a variety of functions other than SG assembly, and thus, the deletion phenotypes may not be explained only by defects in SG assembly.

### Nrd1 is Involved in Recovery from Various Environmental Stresses

**Figure 6:**
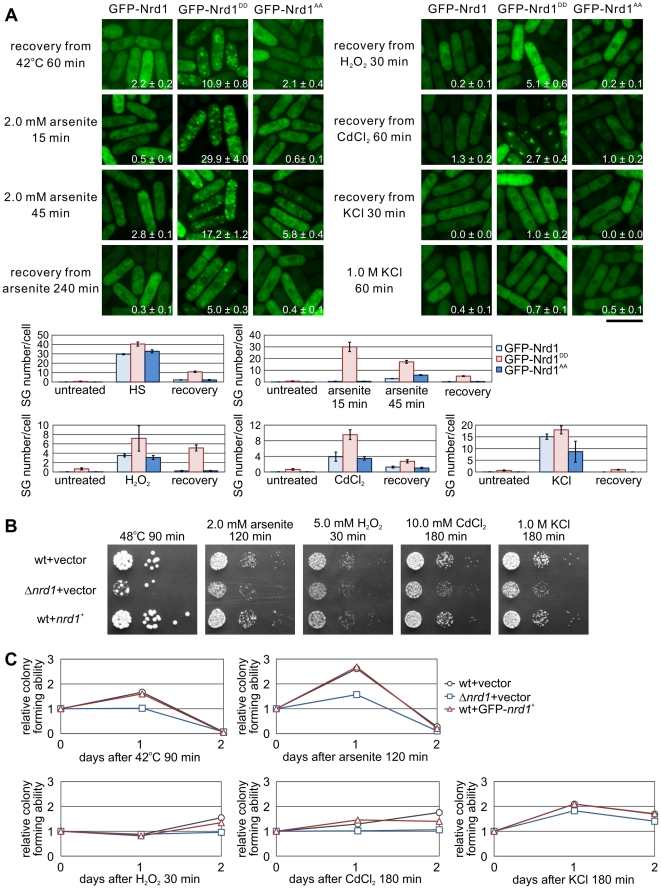
Nrd1 is necessary for recovery from under certain stresses. (A) Disassembly of stress-induced Nrd1 granules. Wild-type cells expressing GFP-tagged Nrd1 were grown in EMM+thiamine at 27°C. After a 20-min incubation at 42°C, the cells were allowed to recover for 60-min by incubating them at 27°C (recovery from 42°C 60 min). After a 15-min exposure to 2.0 mM arsenite at 27°C (2.0 mM arsenite 15 min), the cells were washed and allowed to recover for 30- (2.0 mM arsenite 45 min) or 240-min (recovery from arsenite 240 min). After a 30-min exposure to 5.0 mM H_2_O_2_ at 27°C, the cells were washed and allowed to recover for 30 min (recovery from H_2_O_2_ 30 min). After a 120-min exposure to 10.0 mM CdCl_2_ at 27°C, the cells were washed and allowed to recover for 60 min (recovery from CdCl_2_ 60 min). After a 10-min exposure to 1.0 M KCl at 27°C, the cells were washed and allowed to recover for 30 min (recovery from KCl 30 min). After a 60-min exposure to 1.0 M KCl, Nrd1-positive granules resolved (1.0 M KCl 60 min). Bar, 10 µm. Lower panel: Graphs showing the number of stress granules per cell from each strain after each condition as indicated. (B) Δ*nrd1* cells displayed transient stress sensitivity. Wild-type cells transformed with control vector or the *nrd1* ^+^ genes, or the Δ*nrd1* cells transformed with control vector were grown to mid-log-phase in EMM+thiamine at 27°C. The indicated cells were then exposed to thermal stress (48°C 90 min), 2.0 mM arsenite for 120 min, 5.0 mM H_2_O_2_ for 30 min, 10.0 mM CdCl_2_ for 180 min, and 1.0 M KCl for 180 min and were then spotted onto YES plates and incubated at 27°C. (C) CPU assay of the cells as indicated in Figure 6(B). Cells were treated as indicated in Figure 6(B), and the colony forming ability of each strain after each condition as indicated was determined by counting the number of viable colonies and normalized to the number of colonies in unstressed condition for each strain. This experiment is representative of two independently performed experiments.

We determined the time required for the disassembly of SGs that were formed when wild-type cells were treated with various stimuli for specific periods of time (that varies depending on the stimuli). We found that after 60 min of recovery from thermal stress (Figure 6A; recovery from 42°C for 60 min), GFP-Nrd1 and GFP-Nrd1^AA^ granules had resolved, whereas Nrd1^DD^ granules persisted longer, with visible fluorescence still observed 60 min after heat shock. In addition, after arsenite treatment for 15 min, cells were washed and allowed to recover from the stress. In cells exposed to sodium arsenite (2.0 mM) for 15 min, translocation to the cytoplasmic granules was more rapid in the case of Nrd1^DD^ than in the wild-type Nrd1 and Nrd1^AA^ (Figure 6A; 2.0 mM arsenite for 15 min), because Nrd1 and Nrd1^AA^ granules were formed 45 min after the exposure of the cells to arsenite (Figure 6A; 2.0 mM arsenite for 45 min). After 240 min of recovery from arsenite stress, GFP-Nrd1^DD^ still formed granules, whereas GFP-Nrd1 and GFP-Nrd1^AA^ had already resolved (recovery from arsenite 240 min). Similarly, in cells exposed to H_2_O_2_, CdCl_2_, and KCl, after 30 min of recovery from H_2_O_2_, 60 min recovery from CdCl_2_, and 30 min recovery from KCl, GFP-Nrd1^DD^ remained within the granules, whereas GFP-Nrd1 and GFP-Nrd1^AA^ granules had almost disappeared. It should be noted that 60 min after the continuous exposure to KCl resolved the Nrd1-positive granules (Figure 6A, 1.0 M KCl 60 min), suggesting that this phenomenon may be functionally related to the sensitivity to KCl associated with Nrd1 deletion cells (Figure 5C).

We then examined whether the deletion and overexpression of Nrd1 would alter the tolerance of the cells to the transient exposure to the thermal stress. The wild-type cells harboring the control vector or the *nrd1* ^+^ genes or the Δ*nrd1* cells transformed with the control vector were exposed to thermal stress (48°C for 90 min), arsenite stress (2.0 mM for 120 min), oxidative stress (5.0 mM H_2_O_2_ for 30 min; or 10.0 mM CdCl_2_ for 180 min), or osmotic stress (1.0 M KCl for 180 min), then incubated at 27°C. As shown in Figure 6B, in contrast to the sustained thermal stress shown in Figure 5C, Nrd1 deletion cells were more sensitive than the wild-type cells to the transient stresses. In contrast, the growth of the wild-type cells overexpressing the Nrd1 protein was almost similar to that harboring the control vector alone (Figure 6B).

In addition, we performed time-course experiments wherein liquid cultures were subjected to transient stresses and number of colony-forming-units was determined. The results revealed that even though the Nrd1 deletion cells showed a greater sensitivity to heat shock and arsenite, the growth of Nrd1 deletion cells and the wild-type cells were not discernable on H_2_O_2_ treatment (Fig. 6C). Nrd1 deletion cells showed a modest growth defect during their recovery from CdCl_2_ and KCl (Fig. 6C). Based on the data showing stress-dependent phosphorylation of Nrd1, we can hypothesize that the contribution of Nrd1 in SG assembly and stress response is dependent on the nature of stresses. Taken together, these results suggest that Nrd1 granule formation is reversible, and that Nrd1 is required for recovery from heat shock and arsenite treatment.

## Discussion

### Identification of Nrd1 as a component of fission yeast SGs

Here, we have presented several lines of evidence demonstrating that Nrd1 plays a role in SG assembly. Subcellular localization studies have shown that in response to various stresses, Nrd1 localized to the cytoplasmic granules, which partly co-localized with Pabp and contained mRNA (Figure 1). Like mammalian SGs, the assembly of the Nrd1-positive granules was blocked with cycloheximide, which traps mRNAs in polysomes (Figure 1D). Importantly, in Nrd1 deletion cells, the assembly of Pabp-positive granule formation was delayed, and when overproduced, Nrd1 formed granules per se without stimuli. Furthermore, Nrd1 overproduction leads to aggregation of Pabp-positive granules, thus raising the possibility that Nrd1 plays a central role in SG formation. This latter observation was reminiscent of the spontaneous formation of granules with TIA-1/TIAR overproduction [5]. Overexpression of recombinant TIA-1 represses the production of co-expressed reporter genes in the absence of exogenous stress, and endogenous TIA-1/TIAR represses the translation of the TNFα transcripts in the absence of stress [23]. Several protein components of yeast or mammalian SGs cause growth inhibition and/or granule formation when overexpressed [29]. Interestingly, overexpression of Nrd1 induced cell growth arrest (data not shown), suggesting that Nrd1 may play a role in translation repression in addition to a role in mRNA stability [10].

Nrd1 was identified as a negative regulator of differentiation [30], as well as a regulator of cytokinesis [10]; this indicated that Nrd1 is involved in 2 physiological processes. Nrd1 localizes to the dot-like structures in response to glucose deprivation, a situation that induces sexual differentiation [25], in addition to the responses to various environmental stimuli as we have shown in this study. These results suggest that the localization of Nrd1 to SGs is intimately connected to its regulation.

### Role of MAPK-mediated phosphorylation and Cpc2 in Nrd1-mediated SG formation

What is the physiological significance of Nrd1 localization to SGs? In our previous study, we found that Nrd1 binds to its target mRNAs in its unphosphorylated form and that Nrd1^DD^, the phosphorylation mimic version of Nrd1 failed to bind and stabilize Cdc4, one of the target mRNAs of Nrd1; this suggested that Pmk1 MAPK phosphorylation negatively regulates Nrd1 activity [10]. In this study, we showed that, compared to unphosphorylatable Nrd1, Nrd1^DD^ translocates to the cytoplasmic granules more rapidly and is more prone to aggregate. It should be noted, however, that even unphosphorylatable Nrd1^AA^ translocated to the RNA granules, although much later than Nrd1^DD^. Thus, the phosphorylation of Nrd1 by MAPK is not itself essential for Nrd1 localization to granules; instead, it may enhance the characteristics of Nrd1 to self-aggregate or translocate into the granules in response to stress. Recently, Arimoto *et al.* identified RACK1 as a binding partner for MEKK4 (MTK1) and showed the role of RACK in MTK1 activation [31]. Interestingly, stress treatments that caused the formation of SGs resulted in the association of RACK1, but not MTK1, with the granules; this indicated that stress treatment causes sequestration of RACK1 into granules, thereby preventing the activation of MTK1. In this study, we showed that the various stresses that triggered SG formation also stimulated Nrd1 phosphorylation. When Nrd1 was phosphorylated, its RNA-binding activity was reduced and Nrd1 was strongly bound to Cpc2. Environmental stress promotes sequestration of the Nrd1/Cpc2 complex into SGs, which may serve as a platform for the nucleation of Pabp-positive RNA granules. After the stress was resolved, Nrd1 restarted binding and stabilizing target mRNAs required for cytokinesis, and the absence of Nrd1 will affect recovery from stresses. Thus, it would be intriguing to speculate that the phosphorylation-dependent sequestration of Nrd1 to SGs might represent a mechanism to suppress the ability of Nrd1 to bind and stabilize target mRNAs such as Cdc4.

In conclusion, this is the first study to demonstrate the role of Nrd1 in SG assembly and stress tolerance and its control by MAPK and the RACK homologue in fission yeast. Given the remarkable conservation of MAPK and RACK, a similar mechanism may regulate SG formation and stress tolerance in other eukaryotes. Further functional and molecular characterization of Nrd1 function may help in gaining an understanding of how eukaryotic cells integrate signaling information to regulate the mitotic cycle and differentiation.

## Supporting Information

Figure S1 **Nrd1 is involved in stress-induced granule assembly independent of the phosphorylation of eIF2α.** Wild-type, *eIF2α-S52A*, or Δ*gcn2*Δ*hri1*Δ*hri2* cells expressing GFP-tagged Nrd1 were grown in YES medium at 27°C (untreated) and were subjected to a 5- or 10-min incubation at 42°C (42°C 5 min or 10 min) or 120-min incubation to 2.0 mM arsenite at 27°C (2.0 mM arsenite 120 min). Bar, 10 µm.
(TIF)

## References

[1] Guil S, Long JC, Caceres JF (2006). hnRNP A1 relocalization to the stress granules reflects a role in the stress response.. Mol Cell Biol.

[2] Anderson P, Kedersha N (2009). Stress granules.. Curr Biol.

[3] Anderson P, Kedersha N (2006). RNA granules.. J Cell Biol.

[4] Kedersha N, Anderson P (2002). Stress granules: sites of mRNA triage that regulate mRNA stability and translatability.. Biochem Soc Trans.

[5] Kedersha NL, Gupta M, Li W, Miller I, Anderson P (1999). RNA-binding proteins TIA-1 and TIAR link the phosphorylation of eIF-2 alpha to the assembly of mammalian stress granules.. J Cell Biol.

[6] Lopez de Silanes I, Galban S, Martindale JL, Yang X, Mazan-Mamczarz K (2005). Identification and functional outcome of mRNAs associated with RNA-binding protein TIA-1.. Mol Cell Biol.

[7] Buchan JR, Yoon JH, Parker R (2011). Stress-specific composition, assembly and kinetics of stress granules in *Saccharomyces cerevisiae*.. J Cell Sci.

[8] Wen WL, Stevenson AL, Wang CY, Chen HJ, Kearsey SE (2010). Vgl1, a multi-KH domain protein, is a novel component of the fission yeast stress granules required for cell survival under thermal stress.. Nucleic Acids Res.

[9] Nilsson D, Sunnerhagen P (2011). Cellular stress induces cytoplasmic RNA granules in fission yeast.. RNA.

[10] Satoh R, Morita T, Takada H, Kita A, Ishiwata S (2009). Role of the RNA-binding protein Nrd1 and Pmk1 mitogen-activated protein kinase in the regulation of myosin mRNA stability in fission yeast.. Mol Biol Cell.

[11] Tsukahara K, Yamamoto H, Okayama H (1998). An RNA binding protein negatively controlling differentiation in fission yeast.. Mol Cell Biol.

[12] Jeong HT, Ozoe F, Tanaka K, Nakagawa T, Matsuda H (2004). A novel gene, *msa1*, inhibits sexual differentiation in *Schizosaccharomyces pombe*.. Genetics.

[13] Jeong HT, Oowatari Y, Abe M, Tanaka K, Matsuda H (2004). Interaction between a negative regulator (Msa2/Nrd1) and a positive regulator (Cpc2) of sexual differentiation in *Schizosaccharomyces pombe*.. Biosci Biotechnol Biochem.

[14] Moreno S, Klar A, Nurse P (1991). Molecular genetic analysis of fission yeast *Schizosaccharomyces pombe*.. Methods Enzymol.

[15] Toda T, Dhut S, Superti FG, Gotoh Y, Nishida E (1996). The fission yeast *pmk1*
^+^ gene encodes a novel mitogen-activated protein kinase homolog which regulates cell integrity and functions coordinately with the protein kinase C pathway.. Mol Cell Biol.

[16] Bahler J, Wu JQ, Longtine MS, Shah NG, McKenzie A (1998). Heterologous modules for efficient and versatile PCR-based gene targeting in *Schizosaccharomyces pombe*.. Yeast.

[17] Maundrell K (1990). *nmt1* of fission yeast. A highly transcribed gene completely repressed by thiamine.. J Biol Chem.

[18] Tani T, Derby RJ, Hiraoka Y, Spector DL (1995). Nucleolar accumulation of poly (A)^+^ RNA in heat-shocked yeast cells: implication of nucleolar involvement in mRNA transport.. Mol Biol Cell.

[19] Azad AK, Tani T, Shiki N, Tsuneyoshi S, Urushiyama S (1997). Isolation and molecular characterization of mRNA transport mutants in *Schizosaccharomyces pombe*.. Mol Biol Cell.

[20] Kita A, Sugiura R, Shoji H, He Y, Deng L (2004). Loss of Apm1, the µ1 subunit of the clathrin-associated adaptor-protein-1 complex, causes distinct phenotypes and synthetic lethality with calcineurin deletion in fission yeast.. Mol Biol Cell.

[21] Sio SO, Suehiro T, Sugiura R, Takeuchi M, Mukai H (2005). The role of the regulatory subunit of fission yeast calcineurin for in vivo activity and its relevance to FK506 sensitivity.. J Biol Chem.

[22] Brengues M, Parker R (2007). Accumulation of polyadenylated mRNA, Pab1p, eIF4E, and eIF4G with P-bodies in *Saccharomyces cerevisiae*.. Mol Biol Cell.

[23] Kedersha N, Cho MR, Li W, Yacono PW, Chen S (2000). Dynamic shuttling of TIA-1 accompanies the recruitment of mRNA to mammalian stress granules.. J Cell Biol.

[24] Takada H, Nishimura M, Asayama Y, Mannse Y, Ishiwata S (2007). Atf1 is a target of the mitogen-activated protein kinase Pmk1 and regulates cell integrity in fission yeast.. Mol Biol Cell.

[25] Oowatari Y, Jeong H, Tanae K, Nakagawa T, Kawamukai M (2011). Regulation and role of an RNA-binding protein Msa2 in controlling the sexual differentiation of fission yeast.. Curr Genet.

[26] Nunez A, Franco A, Soto T, Vicente J, Gacto M (2010). Fission yeast receptor of activated C kinase (RACK1) ortholog Cpc2 regulates mitotic commitment through Wee1 kinase.. J Biol Chem.

[27] Nunez A, Franco A, Madrid M, Soto T, Vicente J (2009). Role for RACK1 orthologue Cpc2 in the modulation of stress response in fission yeast.. Mol Biol Cell.

[28] Anderson P, Kedersha N (2008). Stress granules: the Tao of RNA triage.. Trends Biochem Sci.

[29] Buchan JR, Parker R (2009). Eukaryotic stress granules: the ins and outs of translation.. Mol Cell.

[30] Yamamoto H, Tsukahara K, Kanaoka Y, Jinno S, Okayama H (1999). Isolation of a mammalian homologue of a fission yeast differentiation regulator.. Mol Cell Biol.

[31] Arimoto K, Fukuda H, Imajoh-Ohmi S, Saito H, Takekawa M (2008). Formation of stress granules inhibits apoptosis by suppressing stress-responsive MAPK pathways.. Nat Cell Biol.

[32] Tvegard T, Soltani H, Skjolberg HC, Krohn M, Nilssen EA (2007). A novel checkpoint mechanism regulating the G1/S transition.. Genes Dev.

[33] Udagawa T, Nemoto N, Wilkinson CR, Narashimhan J, Jiang L (2008). Int6/eIF3e promotes general translation and Atf1 abundance to modulate Sty1 MAPK-dependent stress response in fission yeast.. J Biol Chem.

