# Supplementary figures and images for: Role of the RNA-Binding Protein Nrd1 in Stress Granule Formation and Its Implication in the Stress Response in Fission Yeast
Source: PLoS One. 2012 Jan 19;7(1):e29683. doi: 10.1371/journal.pone.0029683 (PMC3261880; doi:10.1371/journal.pone.0029683)

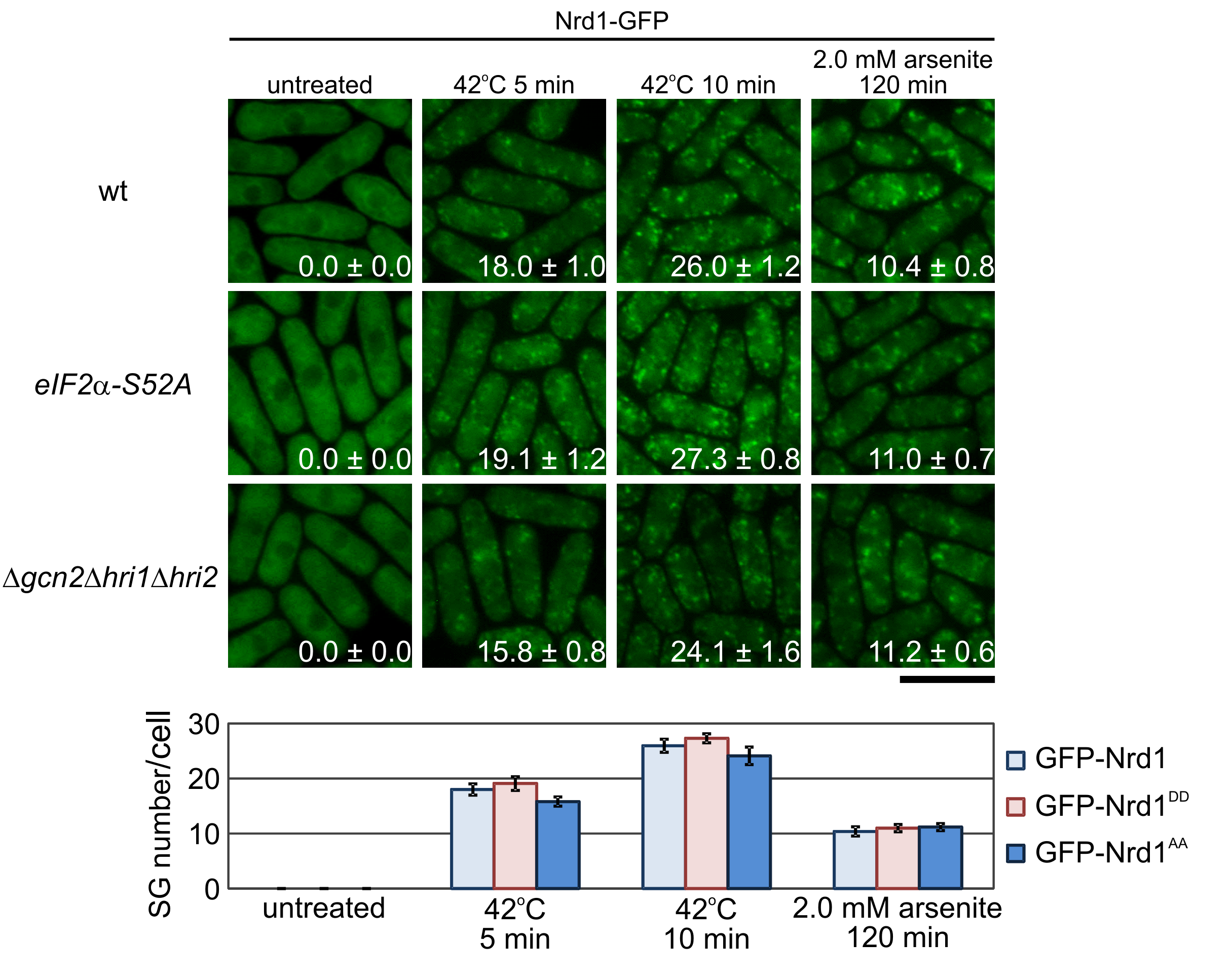

Supplement: Figure S1 — Nrd1 is involved in stress-induced granule assembly independent of the phosphorylation of eIF2α. Wild-type, eIF2α-S52A, or Δgcn2Δhri1Δhri2 cells expressing GFP-tagged Nrd1 were grown in YES medium at 27°C (untreated) and were subjected to a 5- or 10-min incubation at 42°C (42°C 5 min or 10 min) or 120-min incubation to 2.0 mM arsenite at 27°C (2.0 mM arsenite 120 min). Bar, 10 µm. (TIF) [file pone.0029683.s001.tif]
